# Prevalence of tobacco use in urban and rural areas of Pakistan; a sub-study from second National Diabetes Survey of Pakistan (NDSP) 2016 - 2017

**DOI:** 10.12669/pjms.36.4.1705

**Published:** 2020

**Authors:** Abdul Basit, Bilal Bin Younus, Nazish Waris, Asher Fawwad, members NDSP

**Affiliations:** 1 Abdul Basit, FRCP. Professor of Medicine (BMU), Director (BIDE), Baqai Institute of Diabetology and Endocrinology (BIDE), Baqai Medical University (BMU), Karachi, Sindh, Pakistan; 2 Bilal Bin Younus, FRCP. Professor of Medicine, Principal and Associate Dean Academics (FMMDC), Fatima Memorial Medical and Dental College, Lahore, Punjab, Pakistan; 3 Nazish Waris, M.Phil. Clinical Biochemistry and Psychopharmacology Research Unit Department of Biochemistry, University of Karachi, Pakistan. Research Associate, Research Department (BIDE-BMU), Baqai Institute of Diabetology and Endocrinology (BIDE), Baqai Medical University (BMU), Karachi, Sindh, Pakistan; 4 Asher Fawwad, PhD. Professor & Head of the Biochemistry Department (BMU), Honorary Research Director (BIDE), Baqai Institute of Diabetology and Endocrinology (BIDE), Baqai Medical University (BMU), Karachi, Sindh, Pakistan

**Keywords:** Age standardization, Standardized prevalence, Second NDSP, Tobacco, Type-II diabetes

## Abstract

**Objective:**

To assess age standardized prevalence of tobacco use in urban and rural areas of Pakistan.

**Methods:**

This is a sub-study of second National Diabetes Survey of Pakistan (NDSP) 2016-2017. Prevalence of tobacco, ex-tobacco and non-tobacco users was determined in urban/rural areas of four provinces (Punjab, Sindh, Khyber Pakhtunkhwa, and Baluchistan) of Pakistan amongst people aged greater than or equal to 20 years. Information regarding tobacco and non-tobacco users were obtained from second NDSP (2016-2017) predesigned questionnaire. Detailed methodology for demographic, anthropometric and biochemical parameters remained same as reported in second NDSP (2016-2017).

**Results:**

The age-standardized prevalence of tobacco use in Pakistan was found to be 13.4%. Tobacco use in urban areas was 16.3% and rural areas was 11.7%. Tobacco use in urban and rural males was 26.1% and 24.1%, while in females was 7.7% and 3.1%, respectively. The age-standardized prevalence of ex-tobacco use in Pakistan was found to be 2.3%. Ex-tobacco use in urban areas was 2.6% and rural areas was 2.3%. Similarly, ex-tobacco use in urban and rural males was 4.6% and 4.6%, while in females was 0.7% and 0.5%, respectively. Multinomial logistic regression analysis shows that increasing age does not relate towards addiction of tobacco. Males were found to be 7 times (OR 6.94, 95% CI 5.68-8.49) and urban residents twice (OR 2.09, 95% CI 1.73-2.52) more tobacco users than females and those living in rural areas, respectively. From the likelihood ratio test, all variables were found to be statistically significant except for dysglycemia, dyslipidemia and hypertension.

**Conclusion:**

The prevalence of tobacco use is high. As a sub paper of a large national survey, this evidence is expected to serve as an important tool to plan larger studies leading in turn to develop strategies for a successful tobacco control program in the country.

## INTRODUCTION

Tobacco use jeopardizes human health and is one of the major sources for non-communicable diseases including chronic obstructive pulmonary diseases (COPD), cardiovascular diseases, diabetes and cancers.^1^ Tobacco related diseases are a major cause and consequence of poverty in low- and middle-income countries (LMICs).^2^

Globally, 942 million men and 175 million women ages 15 or older smoke tobacco.^3^ It is responsible for death of about seven million people each year; many are premature deaths reported by World Health Organization’s (WHO) new Global Report on Trends in Prevalence of Tobacco Smoking 2000-2025.^4^ However, in developed countries a decline in the prevalence of tobacco use has been reported. Unfortunately, on the contrary, there is increase of tobacco consumption in LMICs. Almost 80% of the world’s smokers live in LMICs and Pakistan is also no exception.^5^ In a recent study, overall prevalence of tobacco use in Pakistan are found less common compared to its neighboring countries (19.1% vs. >25%).^6^

Generally, use of tobacco start around adolescence.^7^ In developing countries, more than half of the adolescents start smoking at an early age and become regular tobacco users subsequently.^8^ Male gender, old age, less education, high coffee or tea consumption, sharing a household with smoker are the most common risk factors for using tobacco.^9^ Tobacco use not only produce various social, cultural, biomedical, economic, and geopolitical problems, but it is also considered to be the key preventable risk factor for morbidity and mortality.^2^

Tobacco control should be a top priority in health issues as well as for reduction of poverty. The Government of Pakistan (GoP) is signatory to the World Health Organization (WHO) and Framework Convention on Tobacco Control (FCTC) that employs six effective strategies and also has also taken a number of initiatives regarding the creation of Tobacco Control Cell at Federal level and health related issues.^6^,^10^,^11^ However, there is a dearth of information on the prevalence, determinants, and management of tobacco use in Pakistan apart from the Global Adult Tobacco Survey (GATS) 2014.^6^ Therefore, this sub-study aimed to assess the age standardized prevalence of tobacco use in urban and rural areas of four provinces of Pakistan amongst people aged 20 years or more.

## METHODS

Data for this sub-study of the primary study was obtained from second National Diabetes Survey of Pakistan (NDSP) 2016-2017, a large, community-based epidemiological survey.^12^ The duration of survey was over 19 months (February 2016 and August 2017). Ethical approval was obtained from the National Bioethics Committee (NBC) of Pakistan (Ref: No.4-87/17/NBC-226/NBC/2664, dated January 31, 2017). Written informed consent was obtained from all participants in their local languages.

The main objective of the survey was to assess the prevalence of Type-II diabetes mellitus (T2DM) and its associated risk factors in urban and rural areas of all four provinces of Pakistan that includes Punjab, Sindh, Khyber Pakhtunkhwa (KPK) and Baluchistan (as defined in latest available census).^12^ Detailed methodology regarding sample size, age standardization, demographic, anthropometric measurements and biochemical analysis were same as in second NDSP (2016-2017).^12^

Information regarding tobacco, ex-tobacco and non-tobacco users for current study were also obtained from the second NDSP (2016-2017) questionnaire. It is a non-comprehensive questionnaire for tobacco assessment as it was mainly designed for the assessment of diabetes. Participants currently using any form of tobacco, irrespective of duration and quantity consumed were called tobacco users, ex-tobacco users as having used tobacco in the past, but not since last month and participants who have never used any form of tobacco were known as non-tobacco users.^12^,^13^ The term tobacco used in our study was defined as the alkaloid nicotine containing compounds mainly used for smoking in manufactured cigarettes, cigars, water pipe (hookah/shisha) and smokeless tobacco (chewing tobacco in the form of paan, gutka, naswar and dipping tobacco).^14^

### Statistical analysis

Statistical analysis were performed using statistical package for social sciences (SPSS Version 22). Estimates were expressed as mean ± SD. Student’s t-test were used to compare groups for continuous variables and Chi square test was used to compare proportions between the two groups. Multiple logistic regression analysis was used to examine the association between outcome variable “tobacco and ex-tobacco users” and various factors like age, gender, place of residence, marital status, education, physical activity, obesity, diabetes, hypertension and dyslipidemia. Using backward selection, variables that remained significant were retained in the final model. P<0.05 was considered statistically significant.

## RESULTS

In second NDSP (2016-2017) survey, 10834 individuals were screened for diabetes fulfilling inclusion criteria. In this sub-study, data regarding tobacco use was available for 9520 (87.9%) participants. The age-standardized prevalence of tobacco use in Pakistan was found to be 13.4%. Tobacco use in urban areas was 16.3% and rural areas was 11.7%. Tobacco use in urban and rural males was 26.1% and 24.1%, while in females was 7.7% and 3.1%, respectively. The age-standardized prevalence of ex-tobacco use in Pakistan was found to be 2.3%. Ex-tobacco use in urban areas was 2.6% and rural areas was 2.3%. Similarly, ex-tobacco use in urban and rural males was 4.6% and 4.6%, while in females was 0.7% and 0.5%, respectively.

Mean age (years) of tobacco users, ex-tobacco users and non-tobacco users was 46.2±13.4, 49.9±13.5 and 43.0±13.7, respectively in urban areas and 48.6±14.7, 50.7±13.2 and 42.8±13.9, in rural areas. Overall, prevalence of tobacco use was higher in province of Baluchistan (34.1%) and (26.5%) followed by Sindh (24.3%) and (19.3%) in both urban and rural areas, respectively, while ex- users were mostly observed in KPK (15.7%) in urban areas and Baluchistan (6.7%) in rural areas. Obesity, hypertension, dysglycemia and dyslipidemia was observed less prevalent in tobacco and ex-tobacco users compared to non-tobacco users (Table-I).

**Table-I:** Baseline and biochemical characteristics of the study participants.

Variables		Urban			Rural			Overall		

		Tobacco user	Ex-tobacco user	Non-tobacco user	Tobacco user	Ex-tobacco user	Non-tobacco user	Tobacco user	Ex-tobacco user	Non-tobacco user
N		592	114	2722	784	163	5145	1376	277	7867
Age (years)		46.2±13.4	49.9±13.5*	43.0±13.7	48.6±14.7	50.7±13.2*	42.8±13.9	47.6±14.2	50.4±13.3*	42.9±13.9
Gender	Male	444 (26.6%)	98 (5.9%)*	1129 (67.6%)	645 (25.6%)	139 (5.5%)*	1732 (68.8%)	1089 (26%)	237 (5.7%)*	2861 (68.3%)
	Female	148 (8.4%)	16 (0.9%)*	1593 (90.7%)	139 (3.9%)	24 (0.7%)*	3413 (95.4%)	287 (5.4%)	40(0.8%)*	5006 (93.9%)
Province	Punjab	168 (10%)	27(1.6%)*	1478 (88.3%)	446 (11.8%)	71 (1.9%)*	3273 (86.4%)	614 (11.2%)	98(1.8%)*	4751 (87%)
	Sindh	306 (24.3%)	20(1.6%)*	931 (74.1%)	187 (19.3%)	20 (2.1%)*	761 (78.6%)	493 (22.2%)	40(1.8%)*	1692 (76%)
	KPK	57 (17.9%)	50(15.7%)*	212 (66.5%)	60 (6.1%)	49 (4.9%)*	882 (89%)	117 (8.9%)	99(7.6%)*	1094 (83.5%)
	Baluchistan	61(34.1%)	17(9.5%)*	101 (56.4%)	91 (26.5%)	23 (6.7%)*	229 (66.8%)	152 (29.1%)	40 (7.7%)*	330 (63.2%)
Marital status	Single	51 (10.8%)	8 (1.7%)*	415 (87.6%)	83 (8.6%)	16 (1.7%)*	869 (89.8%)	134 (9.3%)	24 (1.7%)*	1284 (89%)
	Married	534 (18.6%)	105 (3.7%)*	2228 (77.7%)	681 (13.7%)	146 (2.9%)*	4146 (83.4%)	1215 (15.5%)	251 (3.2%)*	6374 (81.3%)
Body Mass Index (kg/m^2)		26.4±5.4	26.7±4.2*	27.1±5.8	26.3±5.5	26.5±4.5*	27.4±6.3	26.4±5.5	26.6±4.4*	27.3±6.1
WHR		0.94±0.1	0.96±0.16	0.94±0.14	0.91±0.09	0.92±0.1*	0.96±0.24	0.93±0.1	0.95±0.15	0.95±0.19
Education level	Less than primary	176 (20.4%)	19 (2.2%)	668 (77.4%)	455 (13.2%)	76 (2.2%)*	2913 (84.6%)	631 (14.7%)	95 (2.2%)*	3581 (83.1%)
	Primary or more	401 (16.2%)	92 (3.7%)	1986 (80.1%)	298 (12.3%)	85 (3.5%)*	2046 (84.2%)	699 (14.2%)	177(3.6%)*	4032 (82.2%)
Physical activity	No	420 (16.1%)	76 (2.9%)	2107 (80.9%)	482 (10.6%)	116 (2.6%)	3945 (86.8%)	902 (12.6%)	192 (2.7%)	6052 (84.7%)
	Yes	74 (18.6%)	15 (3.8%)	309 (77.6%)	188 (18.6%)	20 (2%)*	803 (79.4%)	262 (18.6%)	35 (2.5%)*	1112 (78.9%)
Alcohol addiction	No	466 (14.9%)	101 (3.2%)	2564 (81.9%)	607 (10.4%)	135 (2.3%)	5082 (87.3%)	1073 (12%)	236 (2.6%)	7646 (85.4%)
	Yes	27 (84.4%)	2 (6.2%)	3(9.4%)*	96 (80%)	8(6.7%)*	16 (13.3%)	123 (80.9%)	10 (6.6%)*	19 (12.5%)
Obesity	No	234 (19.7%)	39 (3.3%)	914 (77%)	274 (14.2%)	53 (2.7%)	1603 (83.1%)	508 (16.3%)	92 (3%)	2517 (80.8%)
	Yes	310 (16.5%)	69 (3.7%)	1496 (79.8%)	401 (12.9%)	94 (3%)	2606 (84%)	711 (14.3%)	163 (3.3%)*	4102 (82.4%)
Dysglycemia	No	365 (16.8%)	78 (3.6%)	1735 (79.7%)	531 (12.7%)	103 (2.5%)	3531 (84.8%)	896 (14.1%)	181 (2.9%)	5266 (83%)
	Yes	227 (18.2%)	36 (2.9%)	987 (79%)	253 (13.1%)	60 (3.1%)	1614 (83.8%)	480 (15.1%)	96 (3%)*	2601 (81.9%)
Hypertension	No	285 (18.5%)	49 (3.2%)	1210 (78.4%)	359 (12.8%)	57(2%)	2380 (85.1%)	644 (14.8%)	106 (2.4%)	3590 (82.7%)
	Yes	300 (17.1%)	60 (3.4%)	1395 (79.5%)	388 (12.7%)	100(3.3%)*	2565 (84%)	688 (14.3%)	160 (3.3%)*	3960 (82.4%)
Dyslipidemia	No	17 (23%)	2 (2.7%)	55 (74.3%)	32 (18.8%)	6(3.5%)	132 (77.6%)	49 (20.1%)	8 (3.3%)	187 (76.6%)
	Yes	457 (17.7%)	51 (2%)	2074 (80.3%)	524 (12.4%)	89(2.1%)*	3620 (85.5%)	981 (14.4%)	140 (2.1%)*	5694 (83.6%)

Data presented as mean ± standard deviation (SD) or n (%), Obesity: BMI≥25 kg/m2

* denotes statistically significant difference at p-value < 0.05 among non-tobacco users, ex-tobacco users and current tobacco users.

**Fig.1:**
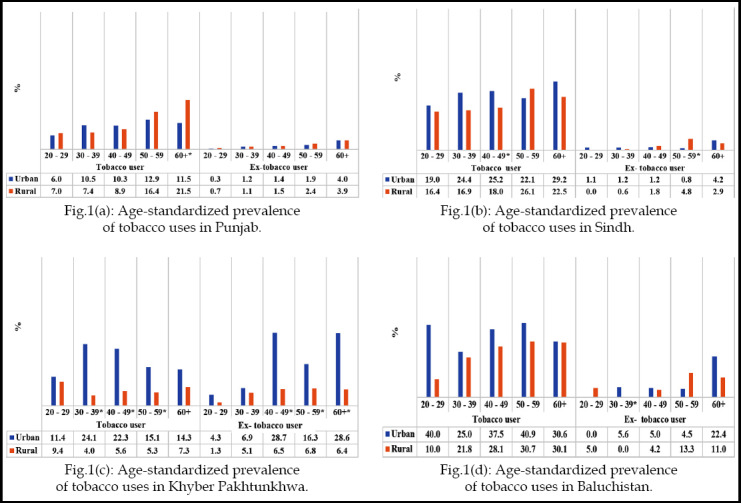
**Fig.1(a):** Age-standardized prevalence of tobacco uses in Punjab **Fig.1(b):** Age-standardized prevalence of tobacco uses in Sindh **Fig.1(c):** Age-standardized prevalence of tobacco uses in Khyber Pakhtunkhwa **Fig.1(d):** Age-standardized prevalence of tobacco uses in Baluchistan

Fig.1 (a-d) shows age-standardized prevalence of tobacco use. In Punjab, rural populations had significantly higher prevalence than urbans in age group ≥ 60 years, while non-significant difference was observed for ex-tobacco users between all age groups (Fig.1(A)). In Sindh, urban had significantly higher prevalence than rural between 40-49 years, while, in ex-tobacco users this trend was higher in rural population between 50-59 years (Fig.1(B)). Urban population of KPK had significantly higher prevalence of tobacco users and ex-tobacco users between 30-59 years and ≥40 years of all age groups, respectively (Fig.1(C)). In Baluchistan, significant results were only observed for urban ex-tobacco users than rural between 30-39 years of age group (Fig.1(D)).

The multinomial logistic regression analysis to assess the association of common and biochemical risk factors associated with tobacco use is shown in Table-II. The OR (95% CI) show that increasing age does not relate towards tobacco use. Males were about seven times more tobacco users than females. Similarly, urban residents about twice more than rural and people having less than primary education about twice more than people having primary education or more were tobacco users. Participants who were about physically less active were found to consume less tobacco. On the other side, significant association was observed with increasing age and ex-tobacco consumption. Males were found to be about 10 times more likely to be an ex- tobacco user than females, married people about twice than single and people having less than primary education 2 times compared to those having education more than primary. From the likelihood ratio test, all variables were found to be statistically significant except for dysglycemia, dyslipidemia and hypertension.

**Table-II:** Odd ratios of univariate and multivariate analysis to assess the association of tobacco use and its associated factors.

Factors		Tobacco users		Ex-tobacco users	

		OR (95% C.I)	P-value	OR (95% C.I)	P-value
Age	20 - 29	0.71(0.49-1.03)	0.072	0.19(0.07-0.52)	0.001
	30 - 39	0.7(0.52-0.94)	.017	0.4(0.2-0.79)	0.009
	40 - 49	0.83(0.64-1.08)	0.162	0.58(0.33-1.02)	0.059
	50 - 59	1.3(1-1.69)	0.047	0.54(0.28-1.03)	0.061
	60+	1		1	
Gender	Male	6.94(5.68-8.49)	<0.0001	10.32(5.87-18.13)	<0.0001
	Female	1		1	
Place of Orientation	Urban	2.09(1.73-2.52)	<0.0001	1.07(0.65-1.74)	0.793
	Rural	1		1	
Marital status	Single	0.75(0.53-1.06)	0.1	1.91(0.93-3.9)	0.077
	Married	1		1	
Education	Less than primary	2.28(1.88-2.78)	<0.0001	2.13(1.32-3.44)	0.002
	Primary or more	1		1	
Activity above average	No	0.66(0.53-0.82)	<0.0001	0.76(0.45-1.29)	0.312
	Yes	1		1	
Obesity WHO	No	1(0.83-1.2)	0.975	0.53(0.33-0.87)	0.012
	Yes	1		1	
Dysglycemia	No	1.01(0.84-1.22)	0.915	1.15(0.73-1.83)	0.539
	Yes	1		1	
Dyslipidemia	No	1.32(0.85-2.07)	0.22	1.56(0.6-4.08)	0.363
	Yes	1		1	
Hypertension	No	1.09(0.9-1.31)	0.383	1.06(0.67-1.68)	0.798
	Yes	1		1	

P-value<0.05 considered to be statistically significant.

## DISCUSSION

This sub-study of nationally representative survey second NDSP (2016-2017) provided the current prevalence of tobacco, ex-tobacco uses and its distribution among adults aged ≥20 years in Pakistan. A key finding of our study indicates the age-standardized prevalence of tobacco and ex-tobacco consumption to be 13.4% and 2.3%, respectively. It shows the declining trend in prevalence of tobacco consumption compared to first national survey in adults GATS- 2014^6^ as well as South-East Asia regions or neighboring countries including Bangladesh and India.^15^ Meanwhile, our results are comparable to Rwanda (12.8%), Tanzania (14.1%), Kenya (13.5%) and some other countries.^16^ In Pakistan, variation of tobacco use at orientation and provincial levels was also observed as it was more prevalent in urban population of Baluchistan followed by Sindh and KPK and rural areas of Punjab.

Various studies have shown high prevalence of tobacco consumption in older age and male gender which support our findings.^17^-^20^ In our study, marked gender difference of tobacco consumption was similar to previous study.^21^ However, tobacco consumption in younger between 20-29 years of age group was more prevalent in only one out of four provinces of Pakistan in our study. Meanwhile, study from China, Mexico and India have shown the highest smoking rates among younger age groups.^19^,^22^ In previous studies, tobacco smoking was associated with lower body weights.^23^ In our study, BMI was significantly higher in non-tobacco users and they were more obese compared to tobacco and ex-tobacco users, similar results were observed in related studies.^24^

Other contributory risk factors such as dysglycemia, dyslipidemia, and hypertension showed no significant association with tobacco and ex-tobacco use in our study similar to study of Khalid N et al., study that showed the 27% cigarette smokers with Type-II diabetes and Ain QU et al. study in which unhealthy relationship between hypertension and smoking was seen.^25^,^26^ In our study, multiple logistic regressions showed that tobacco consumption was not associated with risk for dyslipidemia comparable to Yan-Ling Z et al., study.^27^ On the contrary, previous studies have also confirmed that heavy tobacco smoking is associated with increased total cholesterol and higher risk for abnormal total cholesterol in long-lived subjects.^27^

Use of tobacco mainly cigarette smoking is ever rising in Pakistan.^21^ Increased prevalence was due to accessibility and availability of tobacco that play a critical role in influencing adolescents to use tobacco.^13^ Still, we need to increase awareness programs and stronger implementation to stop tobacco advertisements, tobacco supply, banning free distribution of tobacco products, and events sponsored by tobacco companies, by doing this, we can control this hazard to a larger extent.^26^

### Limitations of the study

Firstly, we lack data regarding tobacco consumption in subjects < 20 years of age group who were not assessed as the cutoff of second NDSP (2016-2017) survey was 20 years. Using a non-comprehensive questionnaire, we are missing smoking and smokeless data separately, smoking related risk factors like peer pressure, witness violence at home, addiction, psychological problems, etc. and also complete tobacco related risk factors details from participants that are established in literature is another limitation.

### Strengths of the study

Strength of our study is provision of age standardized data on prevalence of tobacco and ex-tobacco use in urban and rural population with its contributory biochemical risk factors in this part of the world in a proper epidemiological survey setting.

## CONCLUSION

The prevalence of tobacco use is high. As a sub paper of a large national survey, this evidence is expected to serve as an important tool to plan larger studies leading in turn to develop strategies for a successful tobacco control program in the country.

### Authors’ Contributions

**AB:** Concept, design, interpretation of data, edited and approved the manuscript.

**BBY:** Concept, design and involved in the quality control.

**NW:** Literature search, interpretation of data, wrote the manuscript.

**AF:** Concept, design, interpretation of data, edited and approved the manuscript.

**NDSP Members:** Members were responsible for the supervision of the survey, concept, design, involved in the quality control, data management in their respective areas and responsible for accuracy or integrity of the work. All members approved the final submitted version.
